# Improving the communication of hand hygiene procedures: Controlled observation, redesign, and randomized group comparisons

**DOI:** 10.1017/ice.2020.407

**Published:** 2021-02

**Authors:** Francis T. Durso, Sweta Parmar, Ryan S. Heidish, Skyler Tordoya Henckell, Omer S. Oncul, Jesse T. Jacob

**Affiliations:** 1 School of Psychology, Georgia Institute of Technology, Atlanta, Georgia; 2 Division of Infectious Diseases, Department of Medicine, Emory University, Atlanta, Georgia

## Abstract

**Objective:**

To assess the clarity and efficacy of the World Health Organization (WHO) hand-rub diagram, develop a modified version, and compare the 2 diagrams.

**Design:**

Randomized group design preceded by controlled observation and iterative product redesigns.

**Setting:**

The Cognitive Ergonomics Lab in the School of Psychology at the Georgia Institute of Technology.

**Participants:**

We included participants who were unfamiliar with the WHO hand-rub diagram (convenience sampling) to ensure that performance was based on the diagram and not, for example, on prior experience.

**Methods:**

We iterated through the steps of a human factors design procedure: (1) Participants simulated hand hygiene using ultraviolet (UV) absorbent lotion and a hand-rub technique diagram (ie, WHO or a redesign). (2) Coverage, confusion judgments, and behavioral videos informed potentially improved diagrams. And (3) the redesigned diagrams were compared with the WHO version in a randomized group design. Coverage was assessed across 72 hand areas from multiple UV photographs.

**Results:**

The WHO diagram led to multiple omissions in hand-surface coverage, including inadequate coverage by up to 75% of participants for the ulnar edge. The redesigns improved coverage significantly overall and often substantially.

**Conclusions:**

Human factors modification to the WHO diagram reduced inadequate coverage for naïve users. Implementation of an improved diagram should help in the prevention of healthcare-associated infections.

Hands of healthcare workers (HCWs) have been recognized as a common, critical intermediary in the transfer of pathogens from patient to patient^1,2^; thus, hand hygiene is regularly investigated and audited in hospitals.^3–5^ Hand hygiene research with HCWs has focused on compliance,^2,6,7^ training,^8,9^ amount and concentration of hand rub,^10–13^ and duration^13,14^ using direct observations, microbial counts on the hands, ultraviolet (UV) assessment of hands marked with a fluorescent dye, and fingertip cultures.^2,15^ The widely used World Health Organization (WHO) standard, virtually identical to the European standard (EN1500), has been studied using HCWs and has been compared to other diagrams (eg, Centers for Disease Control and Prevention [CDC] 3-step diagram) and to undirected “responsible application.”^16,17^

Direct observation has found inadequate hand surface coverage in 90% of HCWs.^18^ A UV-marked hand-rub technique using the WHO/EN1500 diagram has shown inadequate coverage of hands in ~30% of HCWs, with the dorsal surface of the hand routinely poorly covered.^16,19–21^ Fingertips, potentially the most contaminated parts of the hand during clinical care,^22^ may be frequently missed during hand hygiene practice.^16,20,21^ All of these findings suggest that inadequate hand surface coverage even by HCWs can result from following standard hand-rub techniques.

Recently, the coronavirus disease 2019 (COVID-19) pandemic made apparent the importance of being able to communicate clearly the appropriate way to clean hands not only to HCWs but also to patients,^23^ families, and the general public. The WHO has listed hand hygiene among the most effective preventive measures for preventing the spread of severe acute respiratory coronavirus virus 2 (SARS-CoV-2) among the general population via both self-contamination and cross contamination from others.^24^ Indeed, many public service efforts during the outbreak have used the WHO diagram (ie, How To HandRub Poster.pdf) to create awareness for proper hand hygiene.

Although some research has been conducted in nonclinical communities on how and when hand hygiene is effective,^25^ including food service,^26^ collegiate,^27,28^ and other educational settings,^29,30^ the effectiveness of the diagrams responsible for communicating proper procedure to the general public has received little attention.

Human factors can play an important role in this public health crisis,^31^ as they have in the past.^32–36^ The goal of the current study was to evaluate and, if necessary, improve the WHO hand-rub diagram using human factors methods and principles to make hand hygiene easier, more natural, safer, and more efficient.

## Methods

### Study design

We used an iterative design approach. Following discussions with infectious disease researchers and practitioners and review of hand hygiene guidelines, we conducted several empirical studies. In phase 1, data were collected on the use of the WHO diagram to identify any problems and, if warranted, to inform its redesign. Phase 2 was a sequence of 2 studies comparing a redesigned diagram to the extant WHO diagram using equal allocation random assignment. Participants in phase 2 were randomly assigned in blocks of 2 participants to either the WHO diagram or a redesigned diagram, using a randomizer.^37^

### Setting and procedure

In a standardized laboratory setting, participants were video recorded as they followed a diagram to simulate cleaning their hands using a gel with superior UV light absorption (Banana Boat 50+ SPF Ultra Sport sunscreen) as a surrogate alcohol-based hand-rub product. After completion, pictures of participants’ hands were taken using a modified Canon EOS RP with a UV filter (Kolari Vision UV Bandpass) and UV flashlights. Coverage was assessed from pictures of several hand positions and angles. Following the simulation, participants ranked up to 4 confusing steps along with explanations of the confusing element.

A lead researcher prepared the randomized assignments and sequenced participant folders in service of a partial blind procedure used to keep awareness of the assigned group from the experimenters until they were required to pull the diagram from the folder.

### Outcomes and analysis

**Fig. 1.**
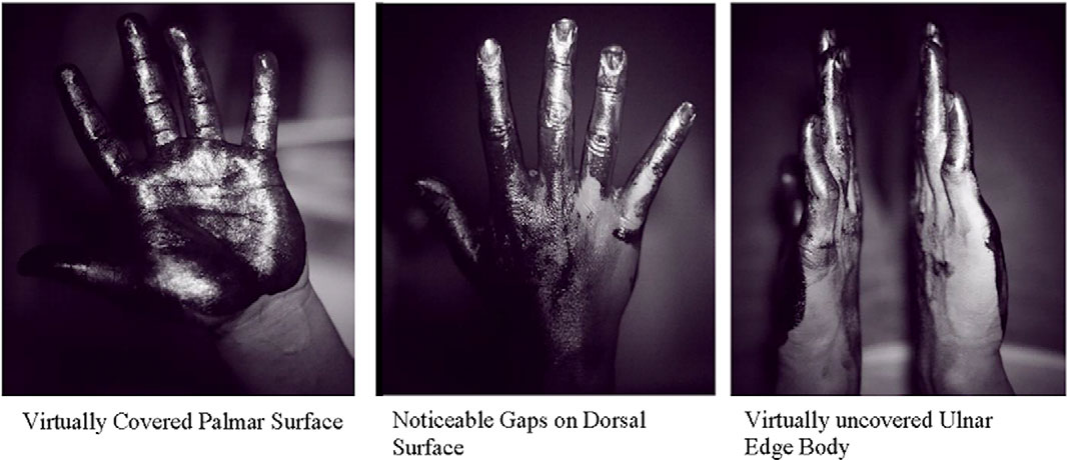
Examples of ultraviolet camera pictures. Dark black color represents sunscreen coverage and bright color represents uncovered areas.

Two human factors researchers, blind to the participant’s group, independently assessed each of 72 surface areas (36 per hand) on a 3-point scale: 0, virtually uncovered; 1, noticeable gaps; or 2, virtually covered (Fig. 1). Reliability between the judges in each study was always >80%.

For the analysis of coverage, we used inadequate coverage scores (the combination of virtually uncovered and noticeable gaps). We computed the proportion of inadequately covered areas for each participant and submitted the data to the nonparametric Mann–Whitney U test. Follow ups looked at the percentage of participants showing inadequate coverage for each hand area graphically.

### Participants

To focus on the diagram as a causal factor in the coverage, rather than previous experience with clinical hand hygiene or with the WHO diagram, we recruited undergraduate volunteers. Participants were from the Georgia Institute of Technology in Atlanta, Georgia; they reported normal or corrected to normal vision.

In phase 1 (June 2019), 30 undergraduates participated in an exploratory study using the WHO diagram alone. In phase 2, the WHO diagram was compared with an initial (phase 2a) and then a final redesign (phase 2b). Power analyses to detect large effects suggested 30 participants per group. Phase 2a took place in the summer of 2019, with 60 undergraduate volunteers assigned randomly to use either the original WHO version or the modified version. We replaced 2 participants due to mechanical difficulties and 1 participant for ignoring the diagram completely. We again sought 60 participants for phase 2b in the spring of 2020. However, data collection was halted on March 13, 2020, when the institute ceased on-campus instruction with the intent to move to remote learning because of the COVID-19 pandemic. Thus, we compared 29 students per group using the original WHO version or the second redesigned version.

### Redesign

Beginning with the results of phase 1 and continuing through phase 2, the redesign process itself began with hand areas that had a large percentage of participants with inadequate coverage. We then traced the area back to the steps in the diagram that were intended to cover that area. For each step, we looked at the videos to evaluate participant compliance and at the participants’ confusion judgments.

## Results

Gender and age information appear in Table 1. The participant sample in each of the 3 studies was typical of the Georgia Tech college population. The 3 correlations among the coverage scores of the WHO groups across studies were all high, confirming successful replication of the WHO exploratory study (Table 2).

**Table 1.** Participant Demographics

Group	No.	Age, y	% Male
Phase 1: WHO	30	20.6	50
Phase 2a: WHO	30	18.9	63
Redesign	30	19.0	50
Phase 2b: WHO	29	19.7	41
Redesign	29	19.6	52

Note. WHO, World Health Organization.

**Table 2.** Correlations (Pearson *r*) Between Inadequate Coverage Across 72 Areas for Each of the WHO Groups Across 3 Phases

Phase	Phase 1: WHO alone	Phase 2a: WHO as Control for 1st Redesign	Phase 2b: WHO as Control for 2^nd^ Redesign
Phase 1		.85	.91
Phase 2a	…	…	.82

Note. WHO, World Health Organization.

### Phase 1: Assessment of the WHO diagram

**Fig. 2.**
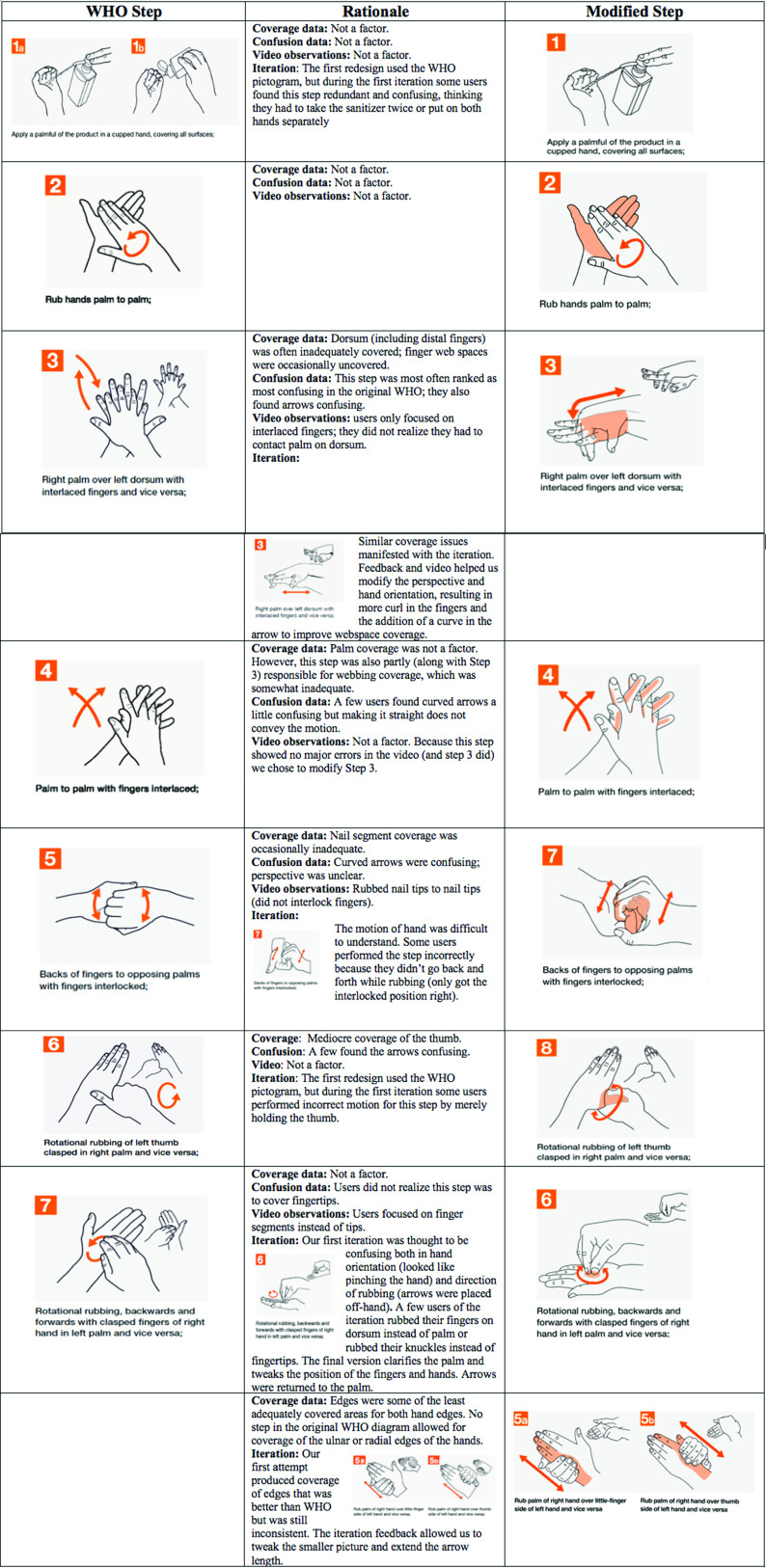
Design modifications and rationale.

Figure 2 shows the WHO diagram steps (column 1), an overview of how the coverage, confusion, and video data informed the redesign process (column 2), and a final redesigned step (column 3). The design rationale relied on the synthesis of these 3 factors.

Coverage of the hand areas varied from exceptional (no participants inadequate) to several areas for which >60% of participants covered inadequately. The poorest coverage was for the ulnar and radial edges of the hands and the dorsum. Because no step in the WHO diagram seemed to address the ulnar and radial edges of the hands, we added new steps 5a and 5b. Several other areas also suggested that there could be room for improvement.

Confusion also varied across the steps, with WHO steps 3, 5, and 7 most often considered confusing. Confusion judgments also alerted us to the particulars of the step that might be problematic (Fig. 2). The videos of hand rubbing often confirmed the participants’ expressed confusion, but they also revealed issues that participants did not report. For example, in step 3, participants did not indicate confusion about rubbing the back of their hands, but they did not perform this action on the video. The videos also suggested that the natural flow of hand rubbing would benefit by moving step 7 in the modified diagram.^14^

**Fig. 3.**
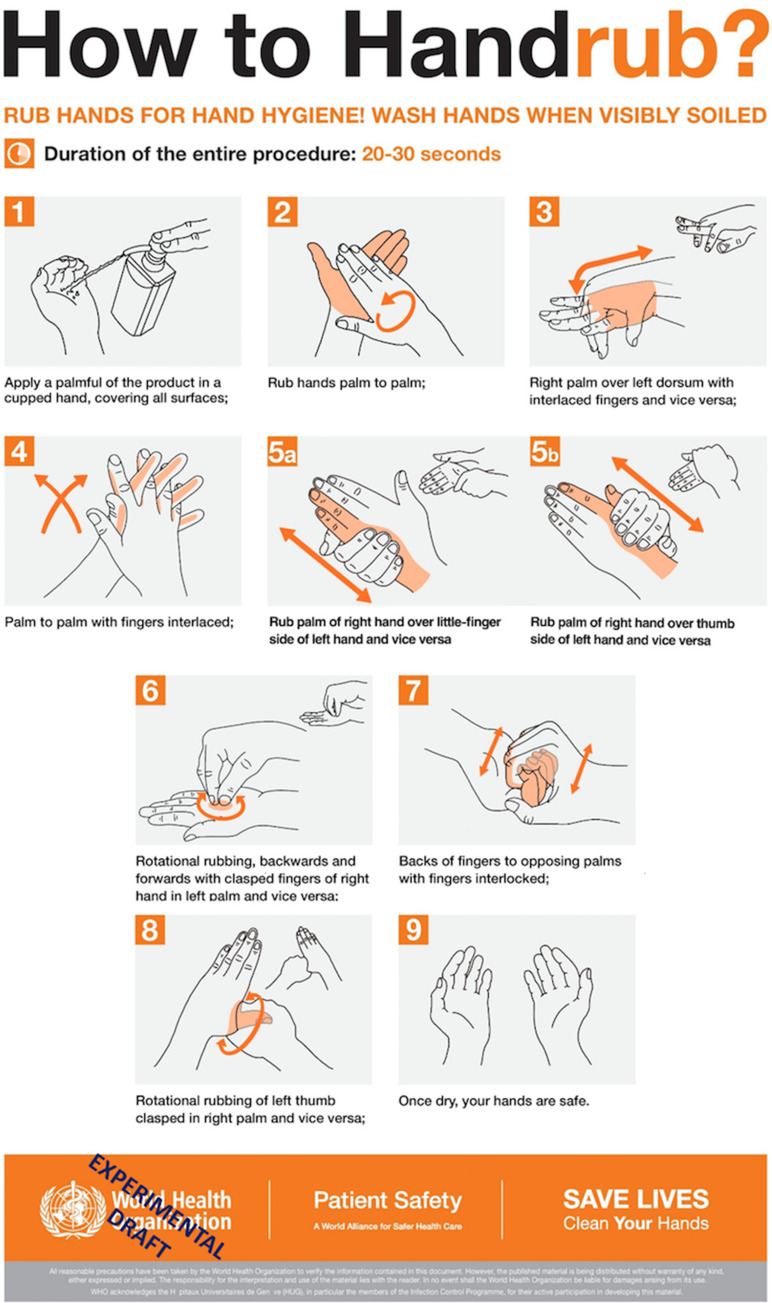
Modification of the WHO diagram based on data from initial exploratory study and the first redesign. This version was compared with the original WHO diagram. Participants saw this diagram without the “experimental draft” overlay.

The design modifications addressed the inadequately covered areas resulting from using the WHO diagram as well as the steps that were especially confusing or were incorrectly performed. The perspective and details of the original WHO depictions were modified to increase clarity. The design modifications were always made on the pictograms; none of the verbiage was changed. The final redesign is reproduced in Figure 3.

### Phase 2a: First redesign evaluation

The redesigned diagram coverage (mean rank, 36.75) was significantly better than the coverage for participants in the WHO group (mean rank, 24.25, Mann–Whitney U = 262.5; *P* = .005; 2-tailed r = .358). Thus, the median redesign participant covered more areas adequately than did the median WHO participant.

Looking at each area, coverage of most hand areas was improved compared to the WHO comparison group. Nevertheless, we felt that a second design iteration could improve coverage further. First, even though the first modification improved areas, coverage of some of those areas had noticeable deficits that we believed could be improved. For example, of the 72 areas, 20 areas showed inadequate coverage in >15% of the participants. Detailed analysis of inadequate coverage along with confusion judgments regarding the steps and videos of the hand rubbing were again used to guide the development of the second design iteration.

In addition to specific problems identified in certain steps, participants did not always understand the area(s) that the steps were intended to cover. Human factors principles suggest that humans are often better if they can perceive a solution rather than think about one. Thus, in the second iteration, we shaded the areas that the step was intended to cover. Some steps required “ghosting” in which a part of the hand, which in reality would be occluded, was made visible (see eg, Fig. 3, step 8). Finally, given the success of the first redesign, we modified additional steps in an effort to reduce further the areas covered inadequately.

### Phase 2b: Final redesign evaluation

Coverage for participants in the redesigned diagram group (mean rank, 33.5) again was significantly better than the coverage for participants in the WHO group (mean rank, 25.50, Mann–Whitney U = 304.5; *P* = .035; 1-tailed, as expected, *r* = .237). Again, the median redesign participant covered fewer areas inadequately than did the median WHO participant. Moreover, 14% of the participants covered all areas adequately using the redesign, compared with 7% for the WHO diagram. The poorest performer covered 60% using the redesign, compared with 40% for the WHO group.

**Fig. 4.**
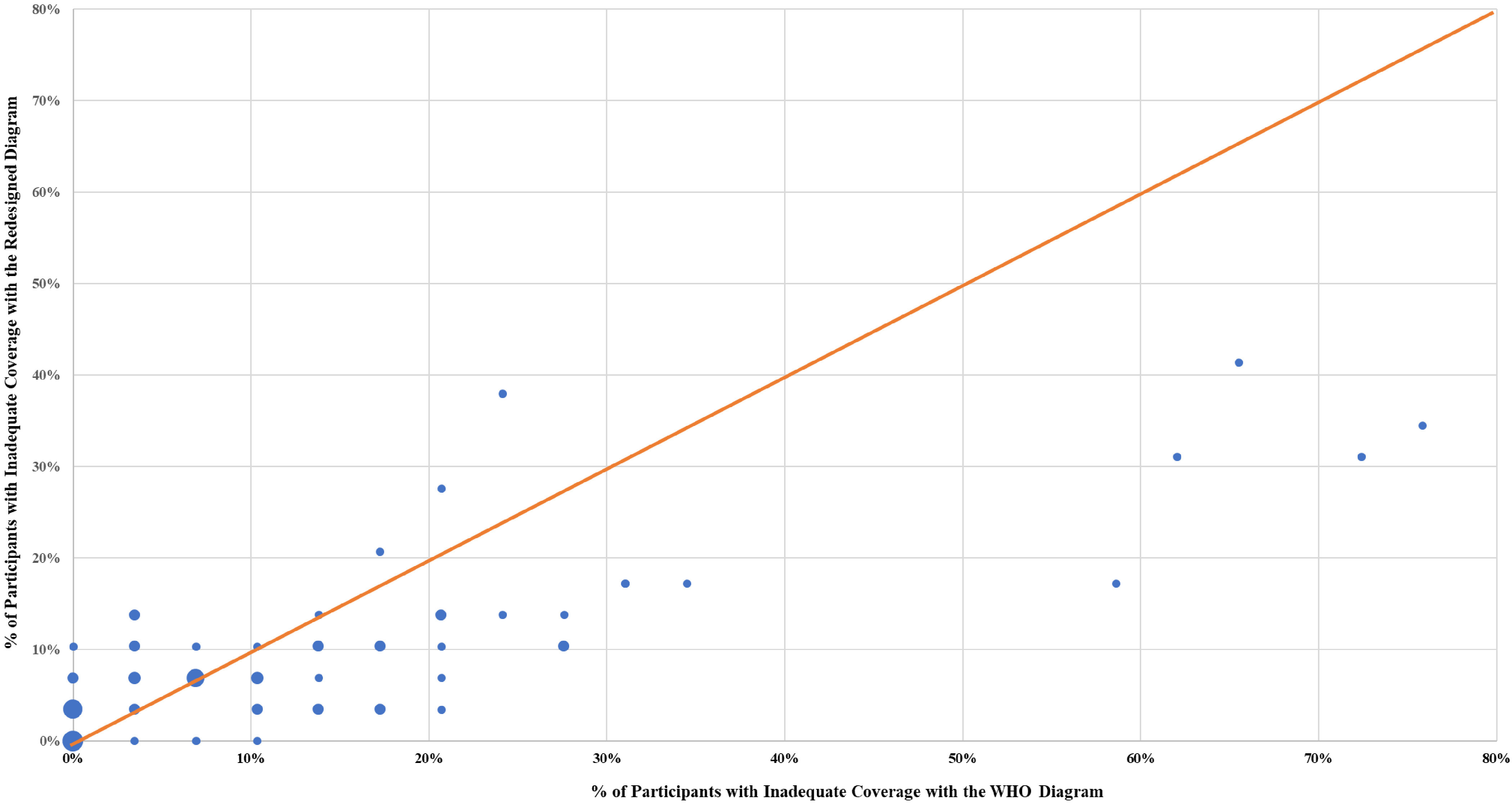
Inadequate coverage for each of the 72 hand areas (% of participants) for the Redesign (ordinate) and WHO (abscissa). Dot diameters reflect number of data points at those coordinates.

Figure 4 conveys an overview of the results of the final redesign by hand area. The abscissa of Figure 4 shows the WHO data, and the ordinate shows the second design iteration data. The line emerging from the origin represents equality. Data points below the line indicate superior performance for the redesigned diagram. Most hand areas were covered adequately by more participants using the redesign than using the WHO diagram. Several hand areas improved over 25 percentage points compared to the WHO version. In addition, the redesign yielded relatively few inadequate areas: In fact, 62 of the 72 hand areas were inadequately covered by <15% of the participants, representing an improvement over the first iteration.

**Fig. 5.**
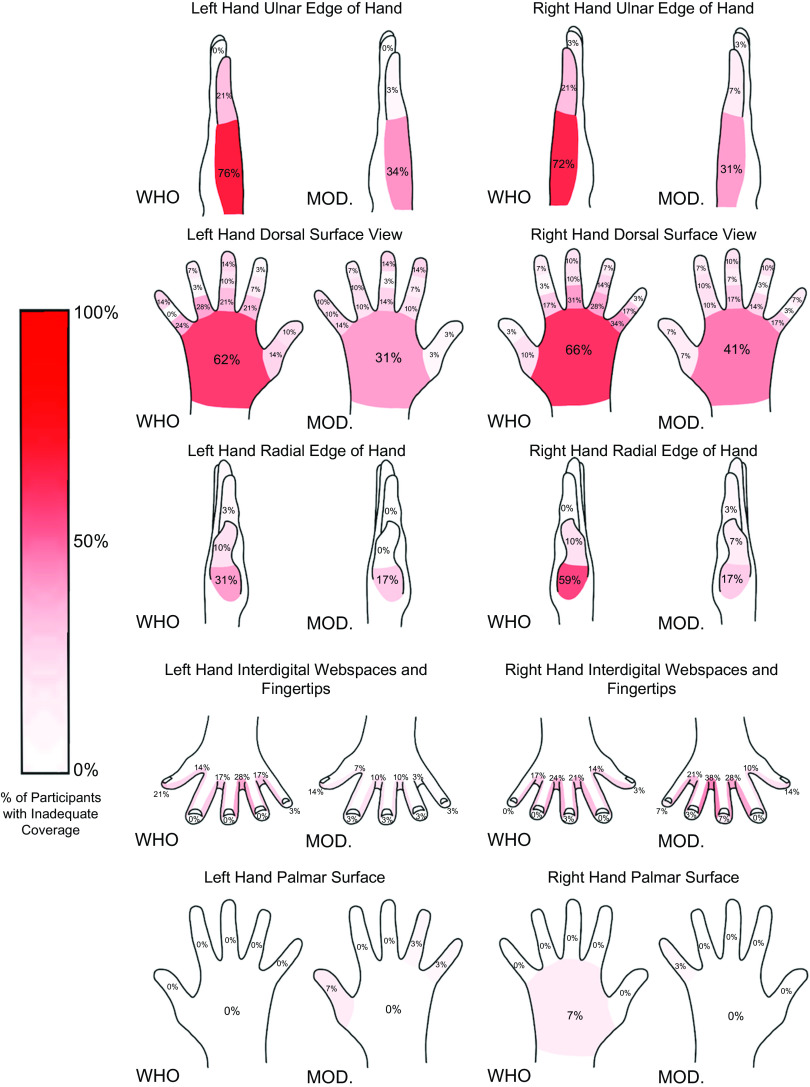
Heat maps of inadequate coverage for both hands and both diagrams. Data represent the percentage of participants who had inadequate coverage (gaps or no coverage) of the indicated area.

Our results do not imply poor performance with the original WHO diagram. In fact, for 30 of the 72 hand areas, all participants, or all but 1, in the WHO group covered adequately (also true for 19 of these areas in the redesign). However, for most of the remaining hand areas, more participants using the redesigned diagram covered completely than those using the WHO diagram. Detailed heat maps (created using the app, Procreate) with the color saturation proportional to the percentage of participants with inadequate coverage data are presented in Figure 5.

#### Ulnar edge of hand

With the WHO diagram, the ulnar edge had the poorest coverage with over 70% of participants covering inadequately. The redesign’s improvement was often substantial, as high as 42 percentage points.

#### Dorsal surface of hand

The dorsal hand coverage was inadequate using the WHO diagram, but it became gradually better as participants moved distally toward the fingertips. Improvement using the redesign was present in multiple comparisons and, again, was often substantial. As participants moved toward the nail tips, the redesign rarely improved over the WHO diagram, which was already performing fairly well.

#### Radial edge of hand

The radial lateral edge of the hand also showed problems with the WHO diagram. The bases of the thumbs were especially poor. The redesign again showed improvement in virtually all of the areas, with improvement as high as 42 percentage points.

#### Interdigital web spaces

Under the WHO diagram, on average, performance was fair and comparable for the 2 hands. This equivalence was not the case with the redesigned diagram. The redesign always outperformed the WHO diagram for the left hand, but virtually never did for the right hand. Further, only 3 hand areas were simultaneously poorer with the redesign and inadequate for >15% of the participants (Fig. 4). All involved the interdigital web spaces of the right hand. The fact that the hands seem to differ in coverage may suggest a difference between dominant and nondominant hands in the performance of the modified step 3.

#### Fingertips

The fingertips were well covered in the WHO and redesign, as discussed earlier. Outperforming the WHO diagram at this level of performance was unlikely.

#### Palmar (or volar) surface of hand

Even more so than the fingertips, coverage of the palm by both diagrams was quite good.

## Discussion

Potential gaps in the baseline WHO diagram were identified in several hand areas. By precise measurement of hand coverage along with procedures to identify difficulties and points of confusion and human factors methods, we were able to design and validate a redesigned diagram showing noticeable improvements in coverage in first time users.

We gained substantial insight for the redesign from the combination of video records, confusion, and coverage. Although coverage was our primary dependent variable driving the design decisions, both participant judgments of confusion and behavioral videos helped in identifying why coverage was bad or good for particular areas, steps, and participants. At times, poorly covered areas were accompanied by indications of confusion. In fact, the redesigned diagram reduced confusion for most of the steps; as one example, the confusion rating for step 8 reduced drastically when redesigned, showing the success of ghosting and highlighting. At other times, some coverage was exemplary even if participants indicated confusion. For example, several participants were confused about the fingertips step in both WHO and the redesigned diagrams, but coverage was quite good. On the other hand, participants might not express confusion,^38^ but the video indicated they continued the step in an incorrect way.

As might be expected with an iterative design procedure, several areas, although improved compared with the original, could be further improved. Surprisingly, although both redesign 1 and redesign 2 showed substantial improvements in the dorsal area of both hands and the ulnar edge of both hands, those 4 areas continue to have a large number of participants who sanitize inadequately (Fig. 4, 4 data points to the far right). We were also perplexed by the only moderate coverage of the web spaces, especially on the right hand and especially between the ring and middle finger, which may be a space physically difficult or awkward to cover. Although the inner web spaces rarely touch a patient or our faces, they might serve as sources of cross contamination, and deserve additional consideration. Notably, complete coverage of all hand surfaces may not be required to prevent contact transmission of infectious diseases, and this ideal might not be achieved with any number of redesign iterations. Adequate coverage of particular hand areas, a goal short of the ideal total coverage of all areas by all individuals, may be sufficient to prevent contact transmission.

Methodological limitations also exist of course. One is that results rest with the inherently subjective nature of human judges evaluating coverage; however, scores indicated high reliability between judges blinded to the diagram assignment, and our results were consistent across phases. The assessment and subsequent modifications of the WHO diagram were based on a relatively small and homogenous group of intelligent individuals presumably focused on and engaged with the task, all limiting generalizability. In addition, generalization should be limited to individuals who actually rely on the diagram to direct their behaviors and not on, for example, their prior experience. Finally, some of the larger benefits from the redesign were due to the addition of a step. Notably, the additional step may cost increased time for hand hygiene, which in turn could lead to lower compliance in field situations. Nevertheless, the redesigned diagram produced significant and sometimes substantial benefits in coverage.

The redesigned diagram can be deployed or it can be used to seed another design iteration. Regardless, the current study has identified clear gaps in the existing WHO diagram’s ability to communicate to the general public and has offered procedures that can be used to investigate and ameliorate those deficits.
